# Fixing and extending some recent results on the ADMM algorithm

**DOI:** 10.1007/s11075-020-00934-5

**Published:** 2020-05-14

**Authors:** Sebastian Banert, Radu Ioan Boţ, Ernö Robert Csetnek

**Affiliations:** 1 Faculty of Engineering (LTH), Department of Automatic Control, Lund University, Box 118, 221 00 Lund, Sweden; 2 Faculty of Mathematics, University of Vienna, Oskar-Morgenstern-Platz 1, A-1090 Vienna, Austria

**Keywords:** ADMM algorithm, Lagrangian, Saddle points, Variable metrics, Positive semidefinite operators, 47H05, 65K05, 90C25

## Abstract

We investigate the techniques and ideas used in Shefi and Teboulle (SIAM J Optim **24**(1), 269–297, 2014) in the convergence analysis of two proximal ADMM algorithms for solving convex optimization problems involving compositions with linear operators. Besides this, we formulate a variant of the ADMM algorithm that is able to handle convex optimization problems involving an additional smooth function in its objective, and which is evaluated through its gradient. Moreover, in each iteration, we allow the use of variable metrics, while the investigations are carried out in the setting of infinite-dimensional Hilbert spaces. This algorithmic scheme is investigated from the point of view of its convergence properties.

## Introduction

One of the most popular numerical algorithms for solving optimization problems of the form

1 $$ \inf_{x\in\mathbb{R}^{n}}\{f(x)+g(Ax)\}, $$

where $f:\mathbb {R}^{n}\rightarrow \overline {\mathbb {R}}:=\mathbb {R}\cup \{\pm \infty \}$ and $g:\mathbb {R}^{m}\rightarrow \overline {\mathbb {R}}$ are proper, convex, lower semicontinuous functions and $A:\mathbb {R}^{n}\rightarrow \mathbb {R}^{m}$ is a linear operator, is the alternating direction method of multipliers (ADMM). Here, the spaces $\mathbb {R}^{n}$ and $\mathbb {R}^{m}$ are equipped with their usual inner products and induced norms, which we both denote by 〈⋅,⋅〉 and ∥⋅∥, respectively, as there is no risk of confusion.

By introducing an auxiliary variable *z*, one can rewrite (1) as

2 $$ \inf_{{(x,z)\in\mathbb{R}^{n}\times\mathbb{R}^{m}\atop Ax-z=0}}\{f(x)+g(z)\}. $$

The Lagrangian associated with problem (2) is

$$l:\mathbb{R}^{n} \times \mathbb{R}^{m} \times \mathbb{R}^{m} \rightarrow \overline{\mathbb{R}}, \ l(x,z,y)=f(x)+g(z)+\langle y,Ax-z\rangle,$$

and we say that $(x^{*},z^{*},y^{*})\in {\mathbb {R}}^{n}\times \mathbb {R}^{m}\times \mathbb {R}^{m}$ is a saddle point of the Lagrangian, if

3 $$ l(x^{*},z^{*},y)\leq l(x^{*},z^{*},y^{*})\leq l(x,z,y^{*}) \ \forall (x,z,y)\in\mathbb{R}^{n}\times\mathbb{R}^{m}\times\mathbb{R}^{m}. $$

It is known that (*x*^∗^, *z*^∗^, *y*^∗^) is a saddle point of *l* if and only if *z*^∗^ = *Ax*^∗^, (*x*^∗^, *z*^∗^) is an optimal solution of (2), *y*^∗^ is an optimal solution of the Fenchel-Rockafellar dual problem (see [3–5, 20, 30]) to (1)

4 $$ \sup_{y\in\mathbb{R}^{m}}\{-f^{*}(-A^{T}y)-g^{*}(y)\}, $$

and the optimal objective values of (1) and (4) coincide. Notice that *f*^∗^ and *g*^∗^ are the conjugates of *f* and *g*, defined by $f^{*}(u)=\sup _{x\in \mathbb {R}^{n}}\{\langle u,x\rangle -f(x)\}$ for all $u \in \mathbb {R}^{n}$ and $g^{*}(y)=\sup _{z\in \mathbb {R}^{m}}\{\langle y,z\rangle -g(z)\}$ for all $y\in \mathbb {R}^{m}$, respectively.

If (1) has an optimal solution and *A*(ri (dom*f*)) ∩ri dom*g*≠*∅*, then the set of saddle points of *l* is nonempty. Here, we denote by ri(*S*) the relative interior of a convex set *S*, which is the interior of *S* relative to its affine hull.

For a fixed real number *c* > 0, we further consider the augmented Lagrangian associated with problem (2), which is defined as

$$L_{c}:\mathbb{R}^{n} \times \mathbb{R}^{m} \times \mathbb{R}^{m} \rightarrow \overline{\mathbb{R}}, \ L_{c}(x,z,y)=f(x)+g(z)+\langle y,Ax-z\rangle + \frac{c}{2} \|Ax-z \|^{2}.$$

The ADMM algorithm reads:

**Figure:**
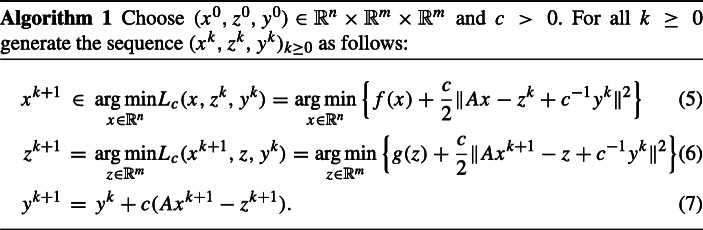

If *A* has full column rank, then the set of minimizers in (5) is a singleton, as is the set of minimizers in (6) without any further assumption, and the sequence (*x*^*k*^, *z*^*k*^, *y*^*k*^)_*k*≥ 0_ generated by Algorithm 1 converges to a saddle point of the Lagrangian *l* (see, for instance, [19]). The alternating direction method of multipliers was first introduced in [25] and [23]. Gabay has shown in [24] (see also [19]) that ADMM is nothing else than the Douglas-Rachford algorithm applied to the monotone inclusion problem

$$0 \in \partial (f^{*} \circ (-A^{T}))(y) + \partial g^{*}(y) $$

For a function $k : \mathbb {R}^{n} \rightarrow \overline {\mathbb {R}}$, the set-valued operator defined by $\partial k(x) := \{u \in \mathbb {R}^{n} : k(t) - k(x) \geq \langle u,t-x\rangle \ \forall t \in \mathcal {R}^{n}\}$, for $k(x) \in \mathbb {R}$, and *∂k*(*x*) := *∅*, otherwise, denotes its (convex) subdifferential.

One of the limitations of the ADMM algorithm comes from the presence of the term *Ax* in the update rule of *x*^*k*+ 1^ (we refer to [14] for an approach to circumvent the limitations of ADMM). While in (6) a proximal step for the function *g* is taken, in (5), the function *f* and the operator *A* are not evaluated independently, which makes the ADMM algorithm less attractive for implementations than the primal-dual splitting algorithms (see, for instance, [8–10, 12, 13, 16, 29]). Despite of this fact, the ADMM algorithm has been widely used for solving convex optimization problems arising in real-life applications (see, for instance, [11, 17, 21]). For a version of the ADMM algorithm with inertial and memory effects, we refer the reader to [7].

In order to overcome the above-mentioned drawback of the classical ADMM method and to increase its flexibility, the following so-called proximal alternating direction proximal method of multipliers has been considered in [28] (see also [22, 26]):

**Figure:**
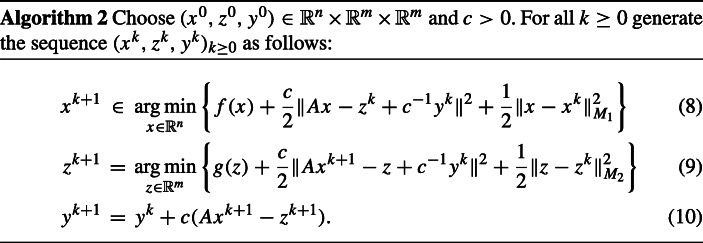

Here, $M_{1}\in \mathbb {R}^{n\times n}$ and $M_{2}\in \mathbb {R}^{m\times m}$ are symmetric positive semidefinite matrices and $\|u\|_{M_{i}}^{2}=\langle u,M_{i}u\rangle $ denotes the squared semi-norm induced by *M*_*i*_, for *i* ∈{1,2}.

Indeed, for *M*_1_ = *M*_2_ = 0, Algorithm 1 becomes the classical ADMM method, while for *M*_1_ = *μ*_1_Id and *M*_2_ = *μ*_2_Id with *μ*_1_, *μ*_2_ > 0 and Id denoting the corresponding matrix, it becomes the algorithm proposed and investigated in [18]. Furthermore, if *M*_1_ = *τ*^− 1^Id − *cA*^*T*^*A* with *τ* > 0 such that *cτ*∥*A*∥^2^ < 1 and *M*_2_ = 0, then one can show that Algorithm 2 is equivalent to one of the primal-dual algorithms formulated in [16].

The sequence (*z*^*k*^)_*k*≥ 0_ generated in Algorithm 2 is uniquely determined due to the fact that the objective function in (9) is lower semicontinuous and strongly convex. On the other hand, the set of minimizers in (8) is in general not a singleton and it can be even empty. However, if one imposes that *M*_1_ + *A*^∗^*A* is positive definite, then (*x*^*k*^)_*k*≥ 0_ will be uniquely determined, too.

Shefi and Teboulle provide in [28] in connection to Algorithm 2 an ergodic convergence rate result for a primal-dual gap function formulated in terms of the Lagrangian *l*, from which they deduce a global convergence rate result for the sequence of function values (*f*(*x*^*k*^) + *g*(*Ax*^*k*^))_*k*≥ 0_ to the optimal objective value of (1), when *g* is Lipschitz continuous. Furthermore, they formulate a global convergence rate result for the sequence (∥*Ax*^*k*^ − *z*^*k*^∥)_*k*≥ 0_ to 0. Finally, Shefi and Teboulle prove the convergence of the sequence (*x*^*k*^, *z*^*k*^, *y*^*k*^)_*k*≥ 0_ to a saddle point of the Lagrangian *l*, provided that either *M*_1_ = 0 and *A* has full column rank or *M*_1_ is positive definite.

Algorithm 2 from [28] represents the starting point of our investigations. More precisely, in this paper:

- We point out some flaws in the proof of a statement in [28], which is fundamental for the derivation of the global convergence rate of (∥*Ax*^*k*^ − *z*^*k*^∥)_*k*≥ 0_ to 0 and of the convergence of the sequence (*x*^*k*^, *z*^*k*^, *y*^*k*^)_*k*≥ 0_.
- We show how the statement in cause can be proved by using different techniques.
- We formulate a variant of Algorithm 2 for solving convex optimization problems in infinite-dimensional Hilbert spaces involving an additional smooth function in their objective that we evaluate through its gradient and which allows in each iteration the use of variable metrics.
- We prove an ergodic convergence rate result for this algorithm involving a primal-dual gap function formulated in terms of the associated Lagrangian *l* and a convergence result for the sequence of iterates to a saddle point of *l*.

## Fixing some results from [28] related to the convergence analysis for Algorithm 2

In this section, we point out several flaws that have been made in [28] when deriving a fundamental result for both the rate of convergence of the sequence (∥*Ax*^*k*^ − *z*^*k*^∥)_*k*≥ 0_ to 0 and the convergence of the sequence (*x*^*k*^, *z*^*k*^, *y*^*k*^)_*k*≥ 0_ to a saddle point of the Lagrangian *l*. We also show how these arguments can be fixed by relying on some of the building blocks of the analysis we will carry out in Section 3.

To proceed, we first recall some results from [28]. We start with a statement that follows from the variational characterization of the minimizers of (8)–(9).

### **Lemma 1**

(see [28, Lemma 4.2]) Let (*x*^*k*^, *z*^*k*^, *y*^*k*^)_*k*≥ 0_ be a sequence generated by Algorithm 2. Then, for all *k* ≥ 0 and for all $(x,z,y)\in \mathbb {R}^{n}\times \mathbb {R}^{m}\times \mathbb {R}^{m}$ it holds

$$ \begin{array}{@{}rcl@{}}l(x^{k+1},z^{k+1},y)& \leq & l(x,z,y^{k+1})+c\langle z^{k+1}-z^{k},A(x-x^{k+1})\rangle\\ &&+\frac{1}{2}\left( \|x - x^{k}\|_{M_{1}}^{2} - \|x - x^{k+1}\|_{M_{1}}^{2} + \|z - z^{k}\|_{M_{2}}^{2} - \|z - z^{k+1}\|_{M_{2}}^{2}\right)\\ &&+\frac{1}{2}\left( c^{-1}\|y-y^{k}\|^{2}-c^{-1}\|y-y^{k+1}\|^{2}\right)\\ &&-\frac{1}{2}\left( \|x^{k+1}-x^{k}\|_{M_{1}}^{2}+\|z^{k+1}-z^{k}\|_{M_{2}}^{2}+c^{-1}\|y^{k+1}-y^{k}\|^{2}\right). \end{array} $$

Furthermore, by invoking the monotonicity of the convex subdifferential of *g*, in [28], the following estimation is derived.

### **Lemma 2**

(see [28, Proposition 5.3(b)]) Let (*x*^*k*^, *z*^*k*^, *y*^*k*^)_*k*≥ 0_ be a sequence generated by Algorithm 2. Then, for all *k* ≥ 1 and for all $(x,z)\in \mathbb {R}^{n}\times \mathbb {R}^{m}$, it holds

$$ \begin{array}{@{}rcl@{}} c\langle z^{k+1}-z^{k},A(x-x^{k+1})\rangle&\leq & \frac{c}{2}\left( \|z-z^{k}\|^{2}-\|z-z^{k+1}\|^{2}+\|Ax-z\|^{2}\right)\\ && +\frac{1}{2}\left( \|z^{k-1}-z^{k}\|_{M_{2}}^{2}-\|z^{k}-z^{k+1}\|_{M_{2}}^{2}\right). \end{array} $$

By taking (*x*,*z*,*y*) := (*x*^∗^, *z*^∗^, *y*^∗^) in Lemma 1, where (*x*^∗^, *z*^∗^, *y*^∗^) is a saddle point of the Lagrangian *l*, and by using the inequality (see (3))

$$l(x^{k+1},z^{k+1},y^{*})\geq l(x^{*},z^{*},y^{k+1}) \ \forall k\geq 0,$$

and the estimation in Lemma 2, one easily obtains the following result.

### **Lemma 3**

Let (*x*^∗^, *z*^∗^, *y*^∗^) be a saddle point of the Lagrangian *l* associated with (1), *M*_1_, *M*_2_ be symmetric positive semidefinite matrices and *c* > 0. Let (*x*^*k*^, *z*^*k*^, *y*^*k*^)_*k*≥ 0_ be a sequence generated by Algorithm 1. Then, for all *k* ≥ 1, the following inequality holds

11 $$ \begin{array}{@{}rcl@{}} && \ \|x^{k+1}-x^{k}\|_{M_{1}}^{2}+\|z^{k+1}-z^{k}\|^{2}_{M_{2}}+c^{-1}\|y^{k+1}-y^{k}\|^{2} \end{array} $$

12 $$ \begin{array}{@{}rcl@{}} &&+ \|x^{*}-x^{k+1}\|_{M_{1}}^{2}+\|z^{*}-z^{k+1}\|_{M_{2}+cI_{m}}^{2}+c^{-1}\|y^{*}-y^{k+1}\|^{2}\\ &&+\|z^{k+1}-z^{k}\|_{M_{2}}^{2} \end{array} $$

13 $$ \begin{array}{@{}rcl@{}} \!\!&\leq &\!\!\|x^{*}-x^{k}\|_{M_{1}}^{2}+\|z^{*}-z^{k}\|_{M_{2}+cI_{m}}^{2}+c^{-1}\|y^{*}-y^{k}\|^{2}+\|z^{k}-z^{k-1}\|_{M_{2}}^{2}. \end{array} $$

By using the notations from [28, Section 5.3], namely

$$v^{k+1}:=\|x^{k+1}-x^{k}\|_{M_{1}}^{2}+\|z^{k+1}-z^{k}\|^{2}_{M_{2}+c\text{Id}}+c^{-1}\|y^{k+1}-y^{k}\|^{2} \ \forall k \geq 0$$

and

$$u^{k}:=\|x^{*}-x^{k}\|_{M_{1}}^{2}+\|z^{*}-z^{k}\|_{M_{2}+c\text{Id}}^{2}+c^{-1}\|y^{*}-y^{k}\|^{2}+\|z^{k}-z^{k-1}\|_{M_{2}}^{2} \ \forall k \geq 1,$$

the inequality in Lemma 3 can be equivalently written as

14 $$ v^{k+1}-c\|z^{k+1}-z^{k}\|^{2}\leq u^{k}-u^{k+1} \ \forall k\geq 1. $$

In [28, Lemma 5.1, (5.37)], instead of (14), it is stated that

15 $$ v^{k+1}\leq u^{k}-u^{k+1} \ \forall k\geq 1, $$

however, its proof, which follows the argument that goes through Lemma 1, Lemma 2, and Lemma 3, is not correct, since it leads to (14) instead of (15).

Since the sequence (*v*^*k*^)_*k*≥ 0_ is monotonically decreasing, statement (15), in combination with straightforward telescoping arguments, leads to the fact that (*v*^*k*^)_*k*≥ 0_ converges to zero with a rate of convergence of $\mathcal {O} (1/k)$. This implies that (∥*Ax*^*k*^ − *z*^*k*^∥)_*k*≥ 0_ converges to zero with a rate of convergence of $\mathcal {O} (1/\sqrt {k})$ (see [28, Theorem 5.4]). In addition, statement (15) is used in [28, Theorem 5.6] to prove the convergence of the sequence (*x*^*k*^, *z*^*k*^, *y*^*k*^)_*k*≥ 0_ to a saddle point of the Lagrangian *l*. However, the techniques used in [28], involving function values and the saddle point inequality, do not lead to (15), but to the weaker inequality (14).

In the following, we will show that one can in fact derive (15), however, to this end one needs to use different techniques. These are described in detail in the next section; here, we will just show how do they lead to (15). We would like to notice that, differently from [28], in our analysis we will only use properties related to the fact that the convex subdifferential of a proper, convex, and lower semicontinuous function is a maximally monotone set-valued operator.

We start our analysis with relation (40), which in case *h* = 0, *L* = 0, ${M_{1}^{k}}=M_{1} \succcurlyeq 0$, and ${M_{2}^{k}}=M_{2} \succcurlyeq 0$ for all *k* ≥ 0 (see the setting of Section 3) reads

16 $$ \begin{array}{@{}rcl@{}} &&c\|z^{k}-Ax^{k+1}\|^{2} + \|x^{k}-x^{k+1}\|_{M_{1}}^{2} + \|z^{k}-z^{k+1}\|_{M_{2}}^{2} \\ &\leq&\|x^{k}-x^{*}\|_{M_{1}}^{2}+\|z^{k}-Ax^{*}\|_{M_{2}+c\text{Id}}^{2}+\frac{1}{c}\|y^{k}-y^{*}\|^{2} \\ &&- \left( \|x^{k+1}-x^{*}\|_{M_{1}}^{2}+\|z^{k+1}-Ax^{*}\|_{M_{2}+c\text{Id}}^{2}+\frac{1}{c}\|y^{k+1}-y^{*}\|^{2}\right). \end{array} $$

for all *k* ≥ 0. Using that

$$ \begin{array}{@{}rcl@{}} c\|z^{k}-Ax^{k+1}\|^{2}& = \ & c \left \|z^{k} - z^{k+1} - \frac{1}{c}(y^{k+1} - y^{k}) \right \|^{2}\\ &= \ & c\|z^{k}-z^{k+1}\|^{2} + \frac{1}{c} \|y^{k+1} - y^{k}\|^{2} + 2 \langle z^{k+1}- z^{k}, y^{k+1} - y^{k}\rangle, \end{array} $$

we obtain from (16) that

17 $$ \begin{array}{@{}rcl@{}} &&2 \langle z^{k+1} - z^{k}, y^{k+1} - y^{k}\rangle + \|x^{k} - x^{k+1}\|_{M_{1}}^{2} + \|z^{k} - z^{k+1}\|_{M_{2}+c\text{Id}}^{2} + \frac{1}{c} \|y^{k+1} - y^{k}\|^{2}  \\ &\leq&\|x^{k}-x^{*}\|_{M_{1}}^{2}+\|z^{k}-Ax^{*}\|_{M_{2}+c\text{Id}}^{2}+\frac{1}{c}\|y^{k}-y^{*}\|^{2} \\ &&- \left( \|x^{k+1}-x^{*}\|_{M_{1}}^{2}+\|z^{k+1}-Ax^{*}\|_{M_{2}+c\text{Id}}^{2}+\frac{1}{c}\|y^{k+1}-y^{*}\|^{2}\right) \end{array} $$

for all *k* ≥ 0. By taking into account that, according to (54),

$$ \begin{array}{@{}rcl@{}} \langle z^{k+1}-z^{k},y^{k+1}-y^{k}\rangle & \geq \frac{1}{2}\|z^{k+1}-z^{k}\|_{M_{2}}^{2}-\frac{1}{2}\|z^{k}-z^{k-1}\|_{M_{2}}^{2} \end{array} $$

for all *k* ≥ 1, it yields

$$ \begin{array}{@{}rcl@{}} &&\|x^{k}-x^{k+1}\|_{M_{1}}^{2} + \|z^{k}-z^{k+1}\|_{M_{2}+c\text{Id}}^{2} + \frac{1}{c} \|y^{k+1} - y^{k}\|^{2} \\ \!& \leq&\! \|x^{k}-x^{*}\|_{M_{1}}^{2}+\|z^{k}-Ax^{*}\|_{M_{2}+c\text{Id}}^{2}+\frac{1}{c}\|y^{k}-y^{*}\|^{2} + \|z^{k} - z^{k-1}\|^{2}_{M_{2}} \\ && - \left( \|x^{k+1} - x^{*}\|_{M_{1}}^{2} + \|z^{k+1} - Ax^{*}\|_{M_{2}+c\text{Id}}^{2}+\frac{1}{c}\|y^{k+1} - y^{*}\|^{2} + \|z^{k+1} - z^{k}\|^{2}_{M_{2}}\right), \end{array} $$

which is nothing else than (15).

From here, by using that *v*^*k*+ 1^ ≤ *v*^*k*^ for all *k* ≥ 0 and straightforward telescoping arguments, it follows immediately that (∥*Ax*^*k*^ − *z*^*k*^∥)_*k*≥ 0_ converges to zero with a rate of $\mathcal {O} (1/\sqrt {k})$.

We will see in the following section that the inequality (40) will play an essential role also in the convergence analysis of the sequence of iterates. When applied to the particular context of the optimization problem (1) and Algorithm 2, Theorem 12 provides a rigorous formulation and a correct and clear proof of the convergence result stated in [28, Theorem 5.6].

## A variant of the ADMM algorithm in the presence of a smooth function and by involving variable metrics

In this section, we propose an extension of the ADMM algorithm considered in [28] that we also investigate from the perspective of its convergence properties. This extension is twofold: on the one hand, we consider an additional convex differentiable function in the objective of the optimization problem (1), which is evaluated in the algorithm through its gradient, and on the other hand, instead of fixed matrices *M*_1_ and *M*_2_, we use different matrices in each iteration. Furthermore, we change the setting to infinite-dimensional Hilbert spaces. We start by describing the problem under investigation:

### **Problem 4**

Let ${\mathscr{H}}$ and $\mathcal { G}$ be real Hilbert spaces, $f:{\mathscr{H}}\rightarrow \overline {\mathbb {R}}$, $g: \mathcal G\rightarrow \overline {\mathbb {R}}$ be proper, convex, and lower semicontinuous functions, $h:{\mathscr{H}}\rightarrow \mathbb {R}$ a convex and Fréchet differentiable function with *L*-Lipschitz continuous gradient (where *L* ≥ 0) and $A:{\mathscr{H}}\rightarrow \mathcal { G}$ a linear continuous operator. The Lagrangian associated with the convex optimization problem

18 $$ \inf_{x\in\mathcal{ H}}\{f(x)+h(x)+g(Ax)\} $$

is

$$l: \mathcal{ H} \times \mathcal{ G} \times \mathcal{ G} \rightarrow \overline{\mathbb{R}}, \ l(x,z,y)=f(x)+h(x)+g(z)+\langle y,Ax-z\rangle.$$

We say that $(x^{*},z^{*},y^{*})\in {\mathscr{H}}\times \mathcal { G}\times \mathcal { G}$ is a saddle point of the Lagrangian *l*, if the following inequalities hold

19 $$ l(x^{*},z^{*},y)\leq l(x^{*},z^{*},y^{*})\leq l(x,z,y^{*}) \ \forall (x,z,y)\in\mathcal{ H}\times\mathcal{ G}\times\mathcal{ G}. $$

Notice that (*x*^∗^, *z*^∗^, *y*^∗^) is a saddle point if and only if *z*^∗^ = *Ax*^∗^, *x*^∗^ is an optimal solution of (18), *y*^∗^ is an optimal solution of the Fenchel-Rockafellar dual problem to (18)

20 $$ (D^{\prime}) \ \ \ \sup_{y\in\mathcal{ G}}\{-(f^{*}{\overline{\mathbb{B}}ox} h^{*})(-A^{*}y)-g^{*}(y)\}, $$

and the optimal objective values of (18) and (20) coincide, where $A^{*}:\mathcal { G}\rightarrow {\mathscr{H}}$ is the adjoint operator defined by 〈*A*^∗^*v*,*x*〉 = 〈*v*,*Ax*〉 for all $(v,x)\in \mathcal { G}\times {\mathscr{H}}$. The infimal convolution $f^{*}{\Box } h^{*}:{\mathscr{H}}\rightarrow \overline {\mathbb {R}}$ is defined by $(f^{*}{\Box } h^{*})(x)=\inf _{u\in {\mathscr{H}}}\{f^{*}(u)+h^{*}(x-u)\}$ for all $x\in {\mathscr{H}}$.

For the reader’s convenience, we discuss some situations which lead to the existence of saddle points. This is for instance the case when (18) has an optimal solution and the Attouch-Brézis qualification condition

21 $$ 0\in\text{sri}(\text{dom} g-A(\text{dom} f)) $$

holds. Here, for a convex set $S\subseteq \mathcal { G}$, we denote by

$$\text{sri} S:=\{x\in S:\cup_{\lambda>0}\lambda(S-x) \ \text{is a closed linear subspace of} \ \mathcal{ G}\}$$

its strong relative interior. Notice that the classical interior is contained in the strong relative interior: $\text {int} \ S\subseteq \text {sri} S$; however, in general, this inclusion may be strict. If $\mathcal { G}$ is finite-dimensional, then for a nonempty and convex set $S \subseteq \mathcal { G}$, one has sri*S* = ri*S*. Considering again the infinite-dimensional setting, we remark that condition (21) is fulfilled if there exists $x^{\prime }\in \text {dom} f$ such that $Ax^{\prime }\in \text {dom} g$ and *g* is continuous at $Ax^{\prime }$.

The optimality conditions for the primal-dual pair of optimization problems (18)-(20) read

22 $$ -A^{*}y-\nabla h(x)\in\partial f(x) \text{ and } y\in\partial g(Ax). $$

This means that if (18) has an optimal solution $x^{*}\in {\mathscr{H}}$ and the qualification condition (21) is fulfilled, then there exists $y^{*}\in \mathcal { G}$, an optimal solution of (20), such that (22) holds and (*x*^∗^, *Ax*^∗^, *y*^∗^) is a saddle point of the Lagrangian *l*. Conversely, if the pair $(x^{*},y^{*})\in {\mathscr{H}}\times \mathcal { G}$ satisfies relation (22), then *x*^∗^ is an optimal solution to (18), *y*^∗^ is an optimal solution to (20) and (*x*^∗^, *Ax*^∗^, *y*^∗^) is a saddle point of the Lagrangian *l*. For further considerations on convex duality, we invite the reader to consult [3–5, 20, 30].

Furthermore, we discuss some conditions ensuring that (18) has an optimal solution. Suppose that (18) is feasible, which means that its optimal objective value is not $+\infty $. The existence of optimal solutions to (18) is guaranteed if, for instance, *f* + *h* is coercive (that is $\lim _{\|x\|\rightarrow \infty }(f+h)(x)=+\infty $) and *g* is bounded from below. Indeed, under these circumstances, the objective function of (18) is coercive and the statement follows via [3, Corollary 11.15]. On the other hand, if *f* + *h* is strongly convex, then the objective function of (18) is strongly convex, too, thus (18) has a unique optimal solution (see [3, Corollary 11.16]).

Some more notations are in order before we state the algorithm for solving Problem 4. We denote by $\mathcal { S_{+}}({\mathscr{H}})$ the family of operators $U:{\mathscr{H}}\rightarrow {\mathscr{H}}$ which are linear, continuous, self-adjoint, and positive semidefinite. For $U\in \mathcal { S_{+}}({\mathscr{H}})$, we consider the semi-norm defined by

$$\|x\|_U^2=\langle x,Ux\rangle \ \forall x\in \mathcal{ H}.$$

We also make use of the Loewner partial ordering defined for $U_{1},U_{2}\in \mathcal { S_{+}}({\mathscr{H}})$ by

$$U_1\succcurlyeq U_2\Leftrightarrow \|x\|_{U_1}^2\geq \|x\|_{U_2}^2 \ \forall x\in \mathcal{ H}.$$

Finally, for *α* > 0, we set

$$\mathcal{ P}_{\alpha}(\mathcal{ H})=\{U\in \mathcal{ S_+}(\mathcal{ H}): U\succcurlyeq \alpha\text{Id} \}.$$

**Figure:**
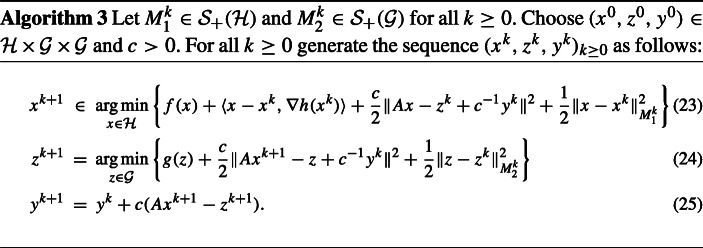

### *Remark 5*

(i) If *h* = 0 and ${M_{1}^{k}}=M_{1}$, ${M_{2}^{k}}=M_{2}$ are constant in each iteration, then Algorithm 3 becomes Algorithm 2, which has been investigated in [28].
(ii) In order to ensure that the sequence (*x*^*k*^)_*k*≥ 0_ is uniquely determined one can assume that for all *k* ≥ 0, there exists ${\alpha _{1}^{k}}>0$ such that ${M_{1}^{k}} + cA^{*}A \in \mathcal { P}_{{\alpha _{1}^{k}}}({\mathscr{H}})$.
  This is in particular the case when
  26 $$ \exists \alpha>0 \text{ such that } A^{*}A\in \mathcal{ P}_{\alpha}(\mathcal{ H}). $$
  Relying on [3, Fact 2.19], on can see that (26) holds if and only if *A* is injective and ran*A*^∗^ is closed. Hence, in finite-dimensional spaces, namely, if ${\mathscr{H}}=\mathbb {R}^{n}$ and $\mathcal { G}=\mathbb {R}^{m}$, with *m* ≥ *n* ≥ 1, (26) is nothing else than saying that *A* has full column rank.
(iii) One of the pioneering works addressing proximal ADMM algorithms in Hilbert spaces, in the particular case when *h* = 0 and ${M_{1}^{k}}$ and ${M_{2}^{k}}$ are equal for all *k* ≥ 0 to the corresponding identity operators, is the paper by Attouch and Soueycatt [2]. We also refer the reader to [22, 26] for versions of the proximal ADMM algorithm stated in finite-dimensional spaces and with proximal terms induced by constant linear operators.

### *Remark 6*

We show that the particular choices ${M_{1}^{k}}=\frac {1}{\tau _{k}}\text {Id}-cA^{*}A$, for *τ*_*k*_ > 0, and ${M_{2}^{k}}=0$ for all *k* ≥ 0 lead to a primal-dual algorithm introduced in [16]. Here, $\text {Id} : {\mathscr{H}} \rightarrow {\mathscr{H}}$ denotes the identity operator on ${\mathscr{H}}$. Let *k* ≥ 0 be fixed. The optimality condition for (23) reads (for *x*^*k*+ 2^):

$$ \begin{array}{@{}rcl@{}}0\!\!&\in&\!\!\partial f(x^{k+2})+cA^{*}(Ax^{k+2}-z^{k+1}\!+c^{-1}y^{k+1})+M_{1}^{k+1}(x^{k+2} - x^{k+1})+\nabla h(x^{k+1})\\ \!\!& = &\!\! \partial f(x^{k+2})+(cA^{*}A+M_{1}^{k+1})x^{k+2}+cA^{*}(-z^{k+1}+c^{-1}y^{k+1})-M_{1}^{k+1}x^{k+1}\\ &&+\nabla h(x^{k+1}). \end{array} $$

From (23), we have

$$cA^{*}(-z^{k+1}+c^{-1}y^{k+1})=A^{*}(2y^{k+1}-y^k)-cA^{*}A x^{k+1};$$

hence,

27 $$ 0\in\partial f(x^{k+2})+(cA^{*}A+M_{1}^{k+1})(x^{k+2}-x^{k+1})+A^{*}(2y^{k+1}-y^{k})+\nabla h(x^{k+1}). $$

By taking into account the special choice of ${M_{1}^{k}}$, we obtain

$$0\in\partial f(x^{k+2})+\frac{1}{\tau_{k+1}}\left( x^{k+2}-x^{k+1}\right)+A^{*}(2y^{k+1}-y^k)+\nabla h(x^{k+1});$$

thus,

28 $$ \begin{array}{@{}rcl@{}} x^{k+2} & = & (\text{Id}+\tau_{k+1}\partial f)^{-1}\left( x^{k+1}-\tau_{k+1}\nabla h(x^{k+1})-\tau_{k+1}A^{*}(2y^{k+1}-y^{k})\right)\\ & = & \underset{x\in \mathcal{ H}}{\arg\min}\left\{f(x)+\frac{1}{2\tau_{k+1}}\left\|x-\left( x^{k+1}-\tau_{k+1}\nabla h(x^{k+1})\right.\right.\right.\\ &&\qquad\qquad \left.\left.\left.-\tau_{k+1}A^{*}(2y^{k+1}-y^{k})\right)\right\|^{2}\right\}. \end{array} $$

Furthermore, from the optimality condition for (24), we obtain

29 $$ c(Ax^{k+1}-z^{k+1}+c^{-1}y^{k})+{M_{2}^{k}}(z^{k}-z^{k+1})\in\partial g(z^{k+1}), $$

which combined with (25) gives

30 $$ y^{k+1}+{M_{2}^{k}}(z^{k}-z^{k+1})\in\partial g(z^{k+1}). $$

Using that ${M_{2}^{k}}=0$ and again (25), it further follows

$$ \begin{array}{@{}rcl@{}}0 &\in& \partial g^{*}(y^{k+1})-z^{k+1}\\ &=& \partial g^{*}(y^{k+1})+c^{-1}(y^{k+1}-y^{k}-cAx^{k+1}), \end{array} $$

which is equivalent to

31 $$ \begin{array}{@{}rcl@{}}y^{k+1} & = &(\text{Id}+c\partial g^{*})^{-1}\left( y^{k}+cAx^{k+1}\right)\\ & = & \underset{z\in \mathcal{ G}}{\arg\min}\left\{g^{*}(z)+\frac{1}{2c}\left\|z-\left( y^{k}+cAx^{k+1}\right)\right\|^{2}\right\}. \end{array} $$

The iterative scheme obtained in (31) and (28) generates, for a given starting point $(x^{1},y^{0})\in {\mathscr{H}} \times \mathcal { G}$ and *c* > 0, the sequence (*x*^*k*^, *y*^*k*^)_*k*≥ 1_ for all *k* ≥ 0 as follows

$$ \begin{array}{@{}rcl@{}} y^{k+1} & = & \underset{z\in \mathcal{G}}{\arg\min}\left\{g^{*}(z)+\frac{1}{2c}\left\|z-\left( y^{k}+cAx^{k+1}\right)\right\|^{2}\right\}\\ x^{k+2} & = & \underset{x\in \mathcal{ H}}{\arg\min}\left\{f(x)+\frac{1}{2\tau_{k+1}}\left\|x-\left( x^{k+1}-\tau_{k+1}\nabla h(x^{k+1})\right.\right.\right.\\ &&\qquad\qquad \left.\left.\left. -\tau_{k+1}A^{*}(2y^{k+1}-y^{k})\right)\right\|^{2}\right\}. \end{array} $$

For *τ*_*k*_ = *τ* > 0 for all *k* ≥ 1, one recovers a primal-dual algorithm from [16] that has been investigated under the assumption $\frac {1}{\tau }-c\|A\|^{2}>\frac {L}{2}$ (see Algorithm 3.2 and Theorem 3.1 in [16]). We invite the reader to consult [8, 9, 13, 29] for more insights into primal-dual algorithms and their highlights. Primal-dual algorithms with dynamic step sizes have been investigated in [13] and [9], where it has been shown that clever strategies in the choice of the step sizes can improve the convergence behavior.

### Ergodic convergence rates for the primal-dual gap

In this section, we will provide a convergence rate result for a primal-dual gap function formulated in terms of the associated Lagrangian *l*. We start by proving a technical statement (see also [28]).

#### **Lemma 7**

In the context of Problem 4, let (*x*^*k*^, *z*^*k*^, *y*^*k*^)_*k*≥ 0_ be a sequence generated by Algorithm 3. Then, for all *k* ≥ 0 and all $(x,z,y)\in {\mathscr{H}}\times \mathcal { G}\times \mathcal { G}$, the following inequality holds

$$ \begin{array}{@{}rcl@{}}l(x^{k+1},z^{k+1},y) &\leq & \ l(x,z,y^{k+1})+c\langle z^{k+1}-z^{k},A(x-x^{k+1})\rangle\\ &+&\frac{1}{2}\left( \|x-x^{k}\|_{{M_{1}^{k}}}^{2} + \|z-z^{k}\|_{{M_{2}^{k}}}^{2} + c^{-1}\|y-y^{k}\|^{2}\right)\\ &-&\frac{1}{2}\left( \|x-x^{k+1}\|_{{M_{1}^{k}}}^{2}+\|z-z^{k+1}\|_{{M_{2}^{k}}}^{2}+c^{-1}\|y-y^{k+1}\|^{2}\right)\\ &-&\frac{1}{2}\left( \|x^{k+1}-x^{k}\|_{{M_{1}^{k}}}^{2}-L\|x^{k+1}-x^{k}\|^{2}+\|z^{k+1}-z^{k}\|_{{M_{2}^{k}}}^{2}\right.\\ &&\quad\quad \left.+c^{-1}\|y^{k+1}-y^{k}\|^{2}\right). \end{array} $$

Moreover, we have for all *k* ≥ 0

$$c\langle z^{k+1}-z^k,A(x-x^{k+1})\rangle\leq \frac{c}{2}\left( \|Ax-z^k\|^2-\|Ax-z^{k+1}\|^2\right)+\frac{1}{2c}\|y^{k+1}-y^k\|^2.$$

#### *Proof*

We fix *k* ≥ 0 and $(x,z,y)\in {\mathscr{H}}\times \mathcal { G}\times \mathcal { G}$. Writing the optimality conditions for (23), we obtain

32 $$ -\nabla h(x^{k})+cA^{*}(z^{k}-c^{-1}y^{k}-Ax^{k+1})+{M_{1}^{k}}(x^{k}-x^{k+1})\in\partial f(x^{k+1}). $$

From the definition of the convex subdifferential, we derive

33 $$ \begin{array}{@{}rcl@{}}f(x^{k+1})-f(x)& \leq & \langle \nabla h(x^{k})+cA^{*}(-z^{k}+c^{-1}y^{k}+Ax^{k+1})\\ &&+{M_{1}^{k}}(-x^{k}+x^{k+1}),x-x^{k+1} \rangle\\ &=& \langle \nabla h(x^{k}),x-x^{k+1}\rangle+\langle y^{k+1},A(x-x^{k+1})\rangle\\ &&-c\langle z^{k}-z^{k+1},A(x-x^{k+1})\rangle\\ &&+\langle {M_{1}^{k}}(x^{k+1}-x^{k}),x-x^{k+1}\rangle, \end{array} $$

where for the last equality, we used (25).

Furthermore, we claim that

34 $$ h(x^{k+1})-h(x)\leq -\langle \nabla h(x^{k}),x-x^{k+1}\rangle+\frac{L}{2}\|x^{k+1}-x^{k}\|^{2}. $$

Indeed, this follows by applying the convexity of *h* and the descent lemma (see [3, Theorem 18.15 (iii)]):

$$ \begin{array}{@{}rcl@{}} &&h(x)-h(x^{k+1})-\langle\nabla h(x^{k}),x-x^{k+1}\rangle\\ & \geq& h(x^{k})+\langle \nabla h(x^{k}),x-x^{k}\rangle-h(x^{k+1})-\langle \nabla h(x^{k}),x-x^{k+1}\rangle\\ & =& h(x^{k})-h(x^{k+1})+\langle\nabla h(x^{k}),x^{k+1}-x^{k}\rangle\\ & \geq& -\frac{L}{2}\|x^{k+1}-x^{k}\|^{2}. \end{array} $$

By combining (33) and (34), we obtain

35 $$ \begin{array}{@{}rcl@{}} &&(f+h)(x^{k+1}) \leq (f+h)(x)+\langle y^{k+1},A(x - x^{k+1})\rangle-c\langle z^{k}-z^{k+1},A(x - x^{k+1})\rangle \\ & &+\frac{1}{2}\|x-x^{k}\|_{{M_{1}^{k}}}^{2}-\frac{1}{2}\|x-x^{k+1}\|_{{M_{1}^{k}}}^{2}-\frac{1}{2}\|x^{k+1}-x^{k}\|_{{M_{1}^{k}}}^{2}+\frac{L}{2}\|x^{k+1}-x^{k}\|^{2}. \end{array} $$

From the optimality condition for (24), we obtain

36 $$ c(Ax^{k+1}-z^{k+1}+c^{-1}y^{k})+{M_{2}^{k}}(z^{k}-z^{k+1})\in\partial g(z^{k+1}), $$

which, combined with (25), gives

37 $$ y^{k+1}+{M_{2}^{k}}(z^{k}-z^{k+1})\in\partial g(z^{k+1}). $$

From here, we derive the inequality

38 $$ \begin{array}{@{}rcl@{}} g(z^{k+1})-g(z) &\leq & \ \langle -y^{k+1}+{M_{2}^{k}}(z^{k+1}-z^{k}),z-z^{k+1}\rangle\\ &= & -\langle y^{k+1},z-z^{k+1}\rangle+\frac{1}{2}\|z-z^{k}\|_{{M_{2}^{k}}}^{2}-\frac{1}{2}\|z-z^{k+1}\|_{{M_{2}^{k}}}^{2}\\ &&-\frac{1}{2}\|z^{k+1}-z^{k}\|_{{M_{2}^{k}}}^{2}. \end{array} $$

The first statement of the lemma follows by combining the inequalities (35) and (38) with the identity (see (25))

$$ \begin{array}{@{}rcl@{}} \langle y,Ax^{k+1}-z^{k+1}\rangle&=& \langle y^{k+1},Ax^{k+1}-z^{k+1}\rangle\\ &&+\frac{1}{2c}\left( \|y-y^k\|^2-\|y-y^{k+1}\|^2-\|y^{k+1}-y^k\|^2\right). \end{array} $$

The second statement follows easily from the arithmetic-geometric mean inequality in Hilbert spaces (see [28, Proposition 5.3(a)]). □

A direct consequence of the two inequalities in Lemma 7 is the following result.

#### **Lemma 8**

In the context of Problem 4, assume that ${M_{1}^{k}}-L\text {Id}\in \mathcal { S_{+}}({\mathscr{H}}), {M_{1}^{k}}\succcurlyeq M_{1}^{k+1}$, ${M_{2}^{k}}\in \mathcal { S_{+}}(\mathcal { G}), {M_{2}^{k}}\succcurlyeq M_{2}^{k+1}$ for all *k* ≥ 0, and let (*x*^*k*^, *z*^*k*^, *y*^*k*^)_*k*≥ 0_ be the sequence generated by Algorithm 3. Then, for all *k* ≥ 0 and all $(x,z,y)\in {\mathscr{H}}\times \mathcal { G}\times \mathcal { G}$ the following inequality holds

$$ \begin{array}{@{}rcl@{}}l(x^{k+1},z^{k+1},y)& \leq & l(x,z,y^{k+1})+\frac{c}{2}\left( \|Ax-z^{k}\|^{2}-\|Ax-z^{k+1}\|^{2}\right)\\ &&+\frac{1}{2}\left( \|x-x^{k}\|_{{M_{1}^{k}}}^{2}-\|x-x^{k+1}\|_{M_{1}^{k+1}}^{2}\right.\\ &&\quad \qquad \left.+\|z-z^{k}\|_{{M_{2}^{k}}}^{2}-\|z-z^{k+1}\|_{M_{2}^{k+1}}^{2}\right)\\ &&+\frac{1}{2c}\left( \|y-y^{k}\|^{2}-\|y-y^{k+1}\|^{2}\right). \end{array} $$

We can now state the main result of this subsection.

#### **Theorem 9**

In the context of Problem 4, assume that ${M_{1}^{k}}-L\text {Id}\in \mathcal { S_{+}}({\mathscr{H}}), {M_{1}^{k}}\succcurlyeq M_{1}^{k+1}$, ${M_{2}^{k}}\in \mathcal { S_{+}}(\mathcal { G}), {M_{2}^{k}}\succcurlyeq M_{2}^{k+1}$ for all *k* ≥ 0, and let (*x*^*k*^, *z*^*k*^, *y*^*k*^)_*k*≥ 0_ be the sequence generated by Algorithm 3. For all *k* ≥ 1 define the ergodic sequences

$$\overline x^k:=\frac{1}{k}\sum\limits_{i=1}^{k}x^{i}, \ \overline z^k:=\frac{1}{k}\sum\limits_{i=1}^{k}z^{i}, \ \overline y^k:=\frac{1}{k}\sum\limits_{i=1}^{k}y^{i}.$$

Then for all *k* ≥ 1 and all $(x,z,y)\in {\mathscr{H}}\times \mathcal { G}\times \mathcal { G}$ it holds

$$l(\overline x^k,\overline z^k,y)-l(x,z,\overline y^k)\leq \frac{\gamma(x,z,y)}{k},$$

where $\gamma (x,z,y):= \frac {c}{2}\|Ax-z^{0}\|^{2} +\frac {1}{2}\left (\|x-x^{0}\|_{{M_{1}^{0}}}^{2}+\|z-z^{0}\|_{{M_{2}^{0}}}^{2}\right ) +\frac {1}{2c}\|y-y^{0}\|^{2}. $

#### *Proof*

We fix *k* ≥ 1 and $(x,z,y)\in {\mathscr{H}}\times \mathcal { G}\times \mathcal { G}$. Summing up the inequalities in Lemma 8 for *i* = 0,...,*k* − 1 and using classical arguments for telescoping sums, we obtain

$$ \sum\limits_{i=0}^{k-1}l(x^{k+1},z^{k+1},y)\leq \sum\limits_{i=0}^{k-1}l(x,z,y^{k+1})+\gamma(x,z,y).$$

Since *l* is convex in (*x*,*z*) and linear in *y*, the conclusion follows from the definition of the ergodic sequences. □

#### *Remark 10*

Let (*x*^∗^, *z*^∗^, *y*^∗^) be a saddle point for the Lagrangian *l*. By taking (*x*,*z*,*y*) := (*x*^∗^, *z*^∗^, *y*^∗^) in the above theorem it yields

$$ \begin{array}{@{}rcl@{}} &&(f+h)(\overline x^k)+g(\overline z^k)+\langle y^{*},A\overline x^k-\overline z^k\rangle-\big(f(x^{*}) + h(x^{*}) + g(Ax^{*}) \big)\\ &\leq& \frac{\gamma(x^{*},z^{*},y^{*})}{k} \ \forall k\geq 1, \end{array} $$

where *f*(*x*^∗^) + *h*(*x*^∗^) + *g*(*Ax*^∗^) is the optimal objective value of the problem (18). Hence, if we suppose that the set of optimal solutions of the dual problem (20) is contained in a bounded set, there exists *R* > 0 such that for all *k* ≥ 1

$$ \begin{array}{@{}rcl@{}} &&(f+h)(\overline x^{k})+g(\overline z^{k})+R\|A\overline x^{k}-\overline z^{k}\|-\big(f(x^{*}) + h(x^{*}) + g(Ax^{*}) \big) \\ & \leq& \frac{1}{k} \left( \frac{c}{2}\|Ax^{*}-z^{0}\|^{2} +\frac{1}{2}\|x^{*}-x^{0}\|_{{M_{1}^{0}}}^{2}+\frac{1}{2}\|z^{*}-z^{0}\|_{{M_{2}^{0}}}^{2} +\frac{1}{c}(R^{2}+\|y^{0}\|^{2})\right). \end{array} $$

The set of dual optimal solutions of (20) is equal to the convex subdifferential of the infimal value function of the problem (18)

$$\psi : \mathcal{ G} \rightarrow \overline{\mathbb{R}}, \ \psi(y) = \inf_{x \in \mathcal{ H}} \left( f(x) + h(x) + g(Ax+y) \right),$$

at 0. This set is weakly compact, thus bounded, if 0 ∈int(dom*ψ*) = int(*A*(dom*f*) −dom*g*) (see [3, 5, 30]).

### Convergence of the sequence of generated iterates

In this subsection, we will address the convergence of the sequence of iterates generated by Algorithm 3 (see also [6, Theorem 7]). One of the important tools for the proof of the convergence result will be [15, Theorem 3.3], which we recall below.

#### **Lemma 11**

(see [15, Theorem 3.3]) Let *S* be a nonempty subset of ${\mathscr{H}}$ and (*x*^*k*^)_*k*≥ 0_ a sequence in ${\mathscr{H}}$. Let *α* > 0 and $W^{k}\in \mathcal { P}_{\alpha }({\mathscr{H}})$ be such that $W^{k}\succcurlyeq W^{k+1}$ for all *k* ≥ 0. Assume that:

(i) For all *z* ∈ *S* and for all *k* ≥ 0: $\|x^{k+1}-z\|_{W^{k+1}}\leq \|x^{k}-z\|_{W^{k}}$.
(ii) Every weak sequential cluster point of (*x*^*k*^)_*k*≥ 0_ belongs to *S*.

Then, (*x*^*k*^)_*k*≥ 0_ converges weakly to an element in *S*.

The proof of the convergence result relies on techniques specific to monotone operator theory and does not make use of the values of the objective function or of the Lagrangian *l*. This makes it different from the proofs in [28] and from the majority of other conventional convergence proofs for ADMM methods. To the few exceptions belong [2] and [19].

#### **Theorem 12**

In the context of Problem 4, assume that the set of saddle points of the Lagrangian *l* is nonempty and that ${M_{1}^{k}}-\frac {L}{2}\text {Id}\in \mathcal { S_{+}}({\mathscr{H}}), {M_{1}^{k}}\succcurlyeq M_{1}^{k+1}$, ${M_{2}^{k}}\in \mathcal { S_{+}}(\mathcal { G}), {M_{2}^{k}}\succcurlyeq M_{2}^{k+1}$ for all *k* ≥ 0, and let (*x*^*k*^, *z*^*k*^, *y*^*k*^)_*k*≥ 0_ be the sequence generated by Algorithm 7. If one of the following assumptions

- (I) There exists *α*_1_ > 0 such that ${M_{1}^{k}}-\frac {L}{2}\text {Id}\in \mathcal { P}_{\alpha _{1}}({\mathscr{H}})$ for all *k* ≥ 0.
- (II) There exists *α*,*α*_2_ > 0 such that ${M_{1}^{k}}-\frac {L}{2}\text {Id}+A^{*}A\in \mathcal { P}_{\alpha }({\mathscr{H}})$ and ${M_{2}^{k}}\in \mathcal { P}_{\alpha _{2}}(\mathcal { G})$ for all *k* ≥ 0.
- (III) There exists *α* > 0 such that ${M_{1}^{k}}-\frac {L}{2}\text {Id}+A^{*}A\in \mathcal { P}_{\alpha }({\mathscr{H}})$ and $2M_{2}^{k+1}\succcurlyeq {M_{2}^{k}}\succcurlyeq M_{2}^{k+1}$ for all *k* ≥ 0.

is fulfilled, then (*x*^*k*^, *z*^*k*^, *y*^*k*^)_*k*≥ 0_ converges weakly to a saddle point of the Lagrangian *l*. This means that (*x*^*k*^)_*k*≥ 0_ converges weakly to an optimal solution of problem (18), and (*y*^*k*^)_*k*≥ 0_ converges weakly to an optimal solution of its dual problem (20).

#### *Proof*

Let $S\subseteq {\mathscr{H}}\times \mathcal { G}\times \mathcal { G}$ denote the set of the saddle points of the Lagrangian *l* and (*x*^∗^, *z*^∗^, *y*^∗^) be a fixed element in *S*. Then, *z*^∗^ = *Ax*^∗^ and the optimality conditions hold

$$ -A^{*}y^{*}-\nabla h(x^{*})\in\partial f(x^{*}), \ y^{*}\in\partial g(Ax^{*}). $$

Let *k* ≥ 0 be fixed. Taking into account (32), (36) and the monotonicity of *∂f* and *∂g*, we obtain

$$ \langle cA^{*}(z^k-Ax^{k+1}-c^{-1}y^k)+M_1^k(x^k-x^{k+1})-\nabla h(x^k)+A^{*}y^{*}+\nabla h(x^{*}),x^{k+1}-x^{*}\rangle\!\geq\! 0 $$

and

$$ \langle c(Ax^{k+1}-z^{k+1}+c^{-1}y^k)+M_2^k(z^k-z^{k+1})-y^{*},z^{k+1}-Ax^{*}\rangle\geq 0. $$

We consider first the case *L* > 0. By the Baillon-Haddad Theorem (see [3, Corollary 18.16]), the gradient of *h* is *L*^− 1^-cocoercive; hence, the following inequality holds

$$ \langle\nabla h(x^{*})-\nabla h(x^k),x^{*}-x^k\rangle\geq L^{-1}\|\nabla h(x^{*})-\nabla h(x^k)\|^2. $$

Summing up the three inequalities obtained above, we get

$$ \begin{array}{@{}rcl@{}} &&c\langle z^{k}-Ax^{k+1},Ax^{k+1}-Ax^{*}\rangle+\langle y^{*}-y^{k},Ax^{k+1}-Ax^{*}\rangle\\ &&+\langle\nabla h(x^{*})-\nabla h(x^{k}),x^{k+1}-x^{*}\rangle +\langle {M_{1}^{k}}(x^{k}-x^{k+1}),x^{k+1}-x^{*}\rangle\\ &&+c\langle Ax^{k+1}-z^{k+1},z^{k+1}-Ax^{*}\rangle+\langle y^{k}-y^{*},z^{k+1}-Ax^{*}\rangle \\ &&+\langle {M_{2}^{k}}(z^{k}-z^{k+1}),z^{k+1}-Ax^{*}\rangle+\langle\nabla h(x^{*})-\nabla h(x^{k}),x^{*}-x^{k}\rangle\\ &&-L^{-1}\|\nabla h(x^{*})-\nabla h(x^{k})\|^{2} \geq 0. \end{array} $$

Furthermore, by taking into account (25), it holds

$$ \begin{array}{@{}rcl@{}} x\langle y^{*}-y^k,Ax^{k+1}-Ax^{*}\rangle+\langle y^k-y^{*},z^{k+1}-Ax^{*}\rangle&=&\langle y^{*}-y^k,Ax^{k+1}-z^{k+1}\rangle\\ &=& c^{-1}\langle y^{*}-y^k,y^{k+1}-y^k\rangle. \end{array} $$

By using some expressions of the inner products in terms of norms, we obtain

$$ \begin{array}{@{}rcl@{}} &&\frac{c}{2}\left( \|z^{k}-Ax^{*}\|^{2}-\|z^{k}-Ax^{k+1}\|^{2}-\|Ax^{k+1}-Ax^{*}\|^{2}\right) \\&& + \frac{c}{2}\left( \|Ax^{k+1}-Ax^{*}\|^{2}-\|Ax^{k+1}-z^{k+1}\|^{2}-\|z^{k+1}-Ax^{*}\|^{2}\right) \\ &&+\frac{1}{2c}\left( \|y^{*}-y^{k}\|^{2}+\|y^{k+1}-y^{k}\|^{2}-\|y^{k+1}-y^{*}\|^{2}\right)\\ &&+\frac{1}{2}\left( \|x^{k}-x^{*}\|_{{M_{1}^{k}}}^{2}-\|x^{k}-x^{k+1}\|_{{M_{1}^{k}}}^{2}-\|x^{k+1}-x^{*}\|_{{M_{1}^{k}}}^{2}\right)\\ &&+\frac{1}{2}\left( \|z^{k}-Ax^{*}\|_{{M_{2}^{k}}}^{2}-\|z^{k}-z^{k+1}\|_{{M_{2}^{k}}}^{2}-\|z^{k+1}-Ax^{*}\|_{{M_{2}^{k}}}^{2}\right)\\ &&+\langle\nabla h(x^{*})-\nabla h(x^{k}),x^{k+1}-x^{k}\rangle-L^{-1}\|\nabla h(x^{*})-\nabla h(x^{k})\|^{2} \geq 0. \end{array} $$

By using again relation (25) for expressing *Ax*^*k*+ 1^ − *z*^*k*+ 1^ and by taking into account that

$$ \begin{array}{@{}rcl@{}} &&\langle\nabla h(x^{*})-\nabla h(x^{k}),x^{k+1}-x^{k}\rangle-L^{-1}\|\nabla h(x^{*})-\nabla h(x^{k})\|^{2} \\& =& -L\left\|L^{-1}\left( \nabla h(x^{*})-\nabla h(x^{k})\right)+\frac{1}{2}\left( x^{k}-x^{k+1}\right)\right\|^{2}+\frac{L}{4}\|x^{k}-x^{k+1}\|^{2}, \end{array} $$

it yields

$$ \begin{array}{@{}rcl@{}} &&\frac{1}{2}\|x^{k+1}-x^{*}\|_{{M_{1}^{k}}}^{2}+\frac{1}{2}\|z^{k+1}-Ax^{*}\|_{{M_{2}^{k}}+c\text{Id}}^{2}+\frac{1}{2c}\|y^{k+1}-y^{*}\|^{2} \\& \leq& \frac{1}{2}\|x^{k}-x^{*}\|_{{M_{1}^{k}}}^{2}+\frac{1}{2}\|z^{k}-Ax^{*}\|_{{M_{2}^{k}}+c\text{Id}}^{2}+\frac{1}{2c}\|y^{k}-y^{*}\|^{2} \\ &&-\frac{c}{2}\|z^{k}-Ax^{k+1}\|^{2}-\frac{1}{2}\|x^{k}-x^{k+1}\|_{{M_{1}^{k}}}^{2}-\frac{1}{2}\|z^{k}-z^{k+1}\|_{{M_{2}^{k}}}^{2} \\ &&-L\left\|L^{-1}\left( \nabla h(x^{*})-\nabla h(x^{k})\right)+\frac{1}{2}\left( x^{k}-x^{k+1}\right)\right\|^{2}+\frac{L}{4}\|x^{k}-x^{k+1}\|^{2} \end{array} $$

and from here, by using the monotonicity assumptions on $({M_{1}^{k}})_{k \geq 0}$ and $({M_{2}^{k}})_{k \geq 0}$, we finally get

39 $$ \begin{array}{@{}rcl@{}} &&\frac{1}{2}\|x^{k+1}-x^{*}\|_{M_{1}^{k+1}}^{2}+\frac{1}{2}\|z^{k+1}-Ax^{*}\|_{M_{2}^{k+1}+c\text{Id}}^{2}+\frac{1}{2c}\|y^{k+1}-y^{*}\|^{2} \\ & \leq &\frac{1}{2}\|x^{k}-x^{*}\|_{{M_{1}^{k}}}^{2}+\frac{1}{2}\|z^{k}-Ax^{*}\|_{{M_{2}^{k}}+c\text{Id}}^{2}+\frac{1}{2c}\|y^{k}-y^{*}\|^{2} \\ & &-\frac{c}{2}\|z^{k}-Ax^{k+1}\|^{2}-\frac{1}{2}\|x^{k}-x^{k+1}\|_{{M_{1}^{k}}-\frac{L}{2}\text{Id}}^{2}-\frac{1}{2}\|z^{k}-z^{k+1}\|_{{M_{2}^{k}}}^{2} \\ & &-L\left\|L^{-1}\left( \nabla h(x^{*})-\nabla h(x^{k})\right)+\frac{1}{2}\left( x^{k}-x^{k+1}\right)\right\|^{2}. \end{array} $$

In case *L* = 0, similar arguments lead to the inequality

40 $$ \begin{array}{@{}rcl@{}} &&\frac{1}{2}\|x^{k+1}-x^{*}\|_{M_{1}^{k+1}}^{2}+\frac{1}{2}\|z^{k+1}-Ax^{*}\|_{M_{2}^{k+1}+c\text{Id}}^{2}+\frac{1}{2c}\|y^{k+1}-y^{*}\|^{2} \\ & \leq&\frac{1}{2}\|x^{k}-x^{*}\|_{{M_{1}^{k}}}^{2}+\frac{1}{2}\|z^{k}-Ax^{*}\|_{{M_{2}^{k}}+c\text{Id}}^{2}+\frac{1}{2c}\|y^{k}-y^{*}\|^{2} \\ &&-\frac{c}{2}\|z^{k}-Ax^{k+1}\|^{2}-\frac{1}{2}\|x^{k}-x^{k+1}\|_{{M_{1}^{k}}}^{2}-\frac{1}{2}\|z^{k}-z^{k+1}\|_{{M_{2}^{k}}}^{2}. \end{array} $$

It is easy to see, by using arguments invoking telescoping sums, that, in both cases, (39) and (40) yield

41 $$ \begin{array}{@{}rcl@{}} &&\sum\limits_{k\geq 0}\|z^{k}-Ax^{k+1}\|^{2}<+\infty, \ \sum\limits_{k\geq 0}\|x^{k}-x^{k+1}\|_{{M_{1}^{k}}-\frac{L}{2}\text{Id}}^{2}<+\infty,\\ && \sum\limits_{k\geq 0}\|z^{k}-z^{k+1}\|_{{M_{2}^{k}}}^{2}<+\infty. \end{array} $$

*The case when Assumption (I) is valid*.

By neglecting the negative terms from the right-hand side of both (39) and (40), it follows that the first assumption in Lemma 11 holds, when applied in the product space ${\mathscr{H}}\times \mathcal { G}\times \mathcal { G}$, for the sequence (*x*^*k*^, *z*^*k*^, *y*^*k*^)_*k*≥ 0_, for $W^{k}:= ({M_{1}^{k}},{M_{2}^{k}}+c\text {Id},c^{-1}\text {Id})$ for *k* ≥ 0, and for $S\subseteq {\mathscr{H}}\times \mathcal { G}\times \mathcal { G}$ the set of saddle points of the Lagrangian *l*.

From (41), we get

42 $$ x^{k}-x^{k+1}\rightarrow 0 \ (k\rightarrow+\infty), $$

since ${M_{1}^{k}}-\frac {L}{2}\text {Id}\in \mathcal { P}_{\alpha _{1}}({\mathscr{H}})$ for all *k* ≥ 0 with *α*_1_ > 0, and

43 $$ z^{k}-Ax^{k+1}\rightarrow 0 \ (k\rightarrow+\infty). $$

A direct consequence of (42) and (43) is

44 $$ z^{k}-z^{k+1}\rightarrow 0 \ (k\rightarrow+\infty). $$

From (25), (43), and (44), we derive

45 $$ y^{k}-y^{k+1}\rightarrow 0 \ (k\rightarrow+\infty). $$

The relations (42)–(45) will play an essential role in the verification of the second assumption in Lemma 11. Let $(\overline x,\overline z,\overline y)\in {\mathscr{H}}\times \mathcal { G}\times \mathcal { G}$ be such that there exists (*k*_*n*_)_*n*≥ 0_, $k_{n}\rightarrow +\infty $ (as $n\rightarrow +\infty $), and $(x^{k_{n}},z^{k_{n}}, y^{k_{n}})$ converges weakly to $(\overline x,\overline z,\overline y)$ (as $n\rightarrow +\infty $).

From (42), we obtain that $(Ax^{k_{n}+1})_{n\in \mathbb {N}}$ converges weakly to $A\overline x$ (as $n\rightarrow +\infty $), which combined with (43) yields $\overline z=A\overline x$. We use now the following notations for all *n* ≥ 0

$$ \begin{array}{@{}rcl@{}} a_{n}^{*} &:= & cA^{*}(z^{k_{n}}-Ax^{k_{n}+1}-c^{-1}y^{k_{n}})+M_{1}^{k_{n}}(x^{k_{n}}-x^{k_{n}+1})+\nabla h(x^{k_{n}+1})-\nabla h(x^{k_{n}})\\ a_{n} &:= & x^{k_{n}+1}\\ b_{n}^{*}&:= & y^{k_{n}+1}+M_{2}^{k_{n}}(z^{k_{n}}-z^{k_{n}+1})\\ b_{n}&:= & z^{k_{n}+1}. \end{array} $$

From (32) and (37), we have for all *n* ≥ 0

46 $$ a_{n}^{*}\in\partial (f+h)(a_{n}) $$

and

47 $$ b_{n}^{*}\in\partial g(b_{n}). $$

Furthermore, from (42), we have

48 $$ a_{n} \text{ converges weakly to } \overline x \ (\text{as }n\rightarrow+\infty). $$

From (45) and (44), we obtain

49 $$ b_{n}^{*} \text{ converges weakly to } \overline y \ (\text{as }n\rightarrow+\infty). $$

Moreover, (25) and (45) yield

50 $$ Aa_{n}-b_{n} \text{ converges strongly to } 0 \ (\text{as }n\rightarrow+\infty). $$

Finally, we have

$$ \begin{array}{@{}rcl@{}} a_{n}^{*}+A^{*}b_{n}^{*}&= & \ cA^{*}(z^{k_{n}}-Ax^{k_{n}+1})+A^{*}(y^{k_{n}+1}-y^{k_{n}})+ \ M_{1}^{k_{n}}(x^{k_{n}}-x^{k_{n}+1}) \\ &&+A^{*}M_{2}^{k_{n}}(z^{k_{n}}-z^{k_{n}+1}) + \nabla h(x^{k_{n}+1})-\nabla h(x^{k_{n}}). \end{array} $$

By using the fact that ∇*h* is Lipschitz continuous, from (42)–(45), we get

51 $$ a_{n}^{*}+A^{*}b_{n}^{*} \text{ converges strongly to } 0 \ (\text{as }n\rightarrow+\infty). $$

Taking into account the relations (46)–(51) and applying [1, Proposition 2.4] to the operators *∂*(*f* + *h*) and *∂g*, we conclude that

$$-A^{*}\overline y\in\partial (f+h)(\overline x)=\partial f(\overline x)+\nabla h(\overline x)\text{ and }\overline y\in\partial g(A\overline x);$$

hence, $(\overline x,\overline z,\overline y)=(\overline x,A\overline x,\overline y)$ is a saddle point of the Lagrangian *l*; thus, the second assumption of the Lemma 11 is verified, too. In conclusion, (*x*^*k*^, *z*^*k*^, *y*^*k*^)_*k*≥ 0_ converges weakly to a saddle point of the Lagrangian *l*.

**The case when Assumption (II) is valid**.

We show that the relations (42)–(45) are fulfilled also in this case. Indeed, Assumption (II) allows to derive from (41) that (43) and (44) hold. From (25), (43), and (44), we obtain (45). Finally, the inequalities

$$ \begin{array}{@{}rcl@{}}\alpha\|x^{k+1}-x^{k}\|^{2}\!&\leq &\! \|x^{k+1}-x^{k}\|_{{M_{1}^{k}} - \frac{L}{2}\text{Id}}^{2} + \|Ax^{k+1}-Ax^{k}\|^{2} \\ &\leq &\! \|x^{k+1}-x^{k}\|_{{M_{1}^{k}} - \frac{L}{2}\text{Id}}^{2} + 2\|Ax^{k+1}-z^{k}\|^{2}+2\|z^{k}-Ax^{k}\|^{2} \ \forall k\geq 0\end{array} $$

yield (42).

On the other hand, notice that both (39) and (40) yield

52 $$ \exists\lim_{k\rightarrow+\infty}\left( \frac{1}{2}\|x^{k}-x^{*}\|_{{M_{1}^{k}}}^{2}+ \frac{1}{2}\|z^{k}-z^{*}\|_{{M_{2}^{k}}+c\text{Id}}^{2}+\frac{1}{2c}\|y^{k}-y^{*}\|^{2}\right); $$

hence, (*y*^*k*^)_*k*≥ 0_ and (*z*^*k*^)_*k*≥ 0_ are bounded. Combining this with (25) and the condition imposed on ${M_{1}^{k}}-\frac {L}{2}\text {Id}+A^{*}A$, we derive that (*x*^*k*^)_*k*≥ 0_ is bounded, too. Hence, there exists a weakly convergent subsequence of (*x*^*k*^, *z*^*k*^, *y*^*k*^)_*k*≥ 0_. By using the same arguments as in the proof of (I), it follows that every weak sequential cluster point of (*x*^*k*^, *z*^*k*^, *y*^*k*^)_*k*≥ 0_ is a saddle point of the Lagrangian *l*.

Now, we show that the set of weak sequential cluster points of (*x*^*k*^, *z*^*k*^, *y*^*k*^)_*k*≥ 0_ is a singleton. Let (*x*_1_, *z*_1_, *y*_1_),(*x*_2_, *z*_2_, *y*_2_) be two such weak sequential cluster points. Then, there exist (*k*_*p*_)_*p*≥ 0_,(*k*_*q*_)_*q*≥ 0_, $k_{p}\rightarrow +\infty $ (as $p\rightarrow +\infty $), $k_{q}\rightarrow +\infty $ (as $q\rightarrow +\infty $), a subsequence $(x^{k_{p}},z^{k_{p}}, y^{k_{p}})_{p \geq 0}$ which converges weakly to (*x*_1_, *z*_1_, *y*_1_) (as $p\rightarrow +\infty $), and a subsequence $(x^{k_{q}},z^{k_{q}}, y^{k_{q}})_{q \geq 0}$ which converges weakly to (*x*_2_, *z*_2_, *y*_2_) (as $q\rightarrow +\infty $). As seen, (*x*_1_, *z*_1_, *y*_1_) and (*x*_2_, *z*_2_, *y*_2_) are saddle points of the Lagrangian *l* and *z*_*i*_ = *Ax*_*i*_ for *i* ∈{1,2}. From (52), which is true for every saddle point of the Lagrangian *l*, we derive

53 $$ \exists\lim_{k\rightarrow+\infty}\left( E(x^{k},z^{k},y^{k}; x_{1},z_{1},y_{1})-E(x^{k},z^{k},y^{k}; x_{2},z_{2},y_{2})\right), $$

where, for (*x*^∗^, *z*^∗^, *y*^∗^), the expression *E*(*x*^*k*^, *z*^*k*^, *y*^*k*^;*x*^∗^, *z*^∗^, *y*^∗^) is defined as

$$ E(x^k,z^k,y^k; x^{*},z^{*},y^{*})=\frac{1}{2}\|x^k-x^{*}\|_{M_1^k}^2+ \frac{1}{2}\|z^k-z^{*}\|_{M_2^k+c\text{Id}}^2+\frac{1}{2c}\|y^k-y^{*}\|^2. $$

Further, we have for all *k* ≥ 0

$$ \frac{1}{2}\|x^k-x_1\|_{M_1^k}^2-\frac{1}{2}\|x^k-x_2\|_{M_1^k}^2=\!\frac{1}{2}\|x_2-x_1\|_{M_1^k}^2 +\langle x^k-x_2,M_1^k(x_2-x_1)\rangle, $$

$$ \begin{array}{@{}rcl@{}} \frac{1}{2}\|z^k-z_1\|_{M_2^k+c\text{Id}}^2-\frac{1}{2}\|z^k-z_2\|_{M_2^k+c\text{Id}}^2 &=&\frac{1}{2}\|z_2-z_1\|_{M_2^k+c\text{Id}}^2 \\ &&+\langle z^k-z_2, (M_2^k+c\text{Id})(z_2-z_1)\rangle, \end{array} $$

and

$$ \frac{1}{2c}\|y^k-y_1\|^2-\frac{1}{2c}\|y^k-y_2\|^2=\frac{1}{2c}\|y_2-y_1\|^2 +\frac{1}{c}\langle y^k-y_2, y_2-y_1\rangle. $$

Applying [27, Théorème 104.1], there exists $M_{1}\in \mathcal { S_{+}}({\mathscr{H}})$ such that $({M_{1}^{k}})_{k \geq 0}$ converges to *M*_1_ in the strong operator topology, i.e., $\|{M_{1}^{k}} x - M_{1} x\| \to 0$ for all $x \in {\mathscr{H}}$ (as $k \to +\infty $). Similarly, the monotonicity condition imposed on $({M_{2}^{k}})_{k \geq 0}$ implies that $\sup _{k\geq 0}\|{M_{2}^{k}}+c\text {Id}\|<+\infty $. Thus, according to [15, Lemma 2.3], there exists $\alpha ^{\prime }>0$ and $M_{2}\in \mathcal { P}_{\alpha ^{\prime }}(\mathcal { G})$ such that $({M_{2}^{k}}+c\text {Id})_{k \geq 0}$ converges to *M*_2_ in the strong operator topology (as $k\rightarrow +\infty $).

Taking the limit in (53) along the subsequences (*k*_*p*_)_*p*≥ 0_ and (*k*_*q*_)_*q*≥ 0_ and using the last three relations above, we obtain

$$ \frac{1}{2}\|x_1-x_2\|_{M_1}^2+\langle x_1-x_2,M_1(x_2-x_1)\rangle+ \frac{1}{2}\|z_1-z_2\|_{M_2}^2+\langle z_1-z_2, M_2(z_2-z_1)\rangle $$

$$ +\frac{1}{2c}\|y_1-y_2\|^2+\frac{1}{c}\langle y_1-y_2, y_2-y_1\rangle =\frac{1}{2}\|x_1-x_2\|_{M_1}^2+\frac{1}{2}\|z_1-z_2\|_{M_2}^2+\frac{1}{2c}\|y_1-y_2\|^2; $$

hence,

$$ -\|x_1-x_2\|_{M_1}^2-\|z_1-z_2\|_{M_2}^2-\frac{1}{c}\|y_1-y_2\|^2=0. $$

From here, we get $\|x_{1}-x_{2}\|_{M_{1}} =0$, *z*_1_ = *z*_2_ and *y*_1_ = *y*_2_. Since

$$ \left( \alpha + \frac{L}{2}\right) \|x_1-x_2\|^2 \leq \|x_1-x_2\|_{M_1}^2 + \|Ax_1-Ax_2\|^2, $$

we obtain that *x*_1_ = *x*_2_. In conclusion, (*x*^*k*^, *z*^*k*^, *y*^*k*^)_*k*≥ 0_ converges weakly to a saddle point of the Lagrangian *l*.

**The case when Assumption (III) is valid** Under Assumption (III), we can further refine the inequalities in (39) and (40). Let *k* ≥ 1 be fixed. By considering the relation (37) for consecutive iterates and by taking into account the monotonicity of *∂g*, we derive

$$ \langle z^{k+1}-z^k,y^{k+1}-y^k+M_2^k(z^k-z^{k+1})-M_2^{k-1}(z^{k-1}-z^k)\rangle\geq 0; $$

hence,

54 $$ \begin{array}{@{}rcl@{}} \langle z^{k+1}-z^{k},y^{k+1}-y^{k}\rangle & \geq& \|z^{k+1}-z^{k}\|_{{M_{2}^{k}}}^{2}+\langle z^{k+1}-z^{k},M_{2}^{k-1}(z^{k-1}-z^{k})\rangle \\ & \geq& \|z^{k+1}-z^{k}\|_{{M_{2}^{k}}}^{2}-\frac{1}{2}\|z^{k+1}-z^{k}\|_{M_{2}^{k-1}}^{2}\\ &&-\frac{1}{2}\|z^{k}-z^{k-1}\|_{M_{2}^{k-1}}^{2}. \end{array} $$

Using that *y*^*k*+ 1^ − *y*^*k*^ = *c*(*Ax*^*k*+ 1^ − *z*^*k*+ 1^), the last inequality yields

55 $$ \begin{array}{@{}rcl@{}} &&\|z^{k+1}-z^{k}\|_{{M_{2}^{k}}}^{2}-\frac{1}{2}\|z^{k+1}-z^{k}\|_{M_{2}^{k-1}}^{2}-\frac{1}{2}\|z^{k}-z^{k-1}\|_{M_{2}^{k-1}}^{2} \\ & \leq& \frac{c}{2}\left( \|z^{k}-Ax^{k+1}\|^{2}-\|z^{k+1}-z^{k}\|^{2}-\|Ax^{k+1}-z^{k+1}\|^{2}\right). \end{array} $$

In case *L* > 0, adding (55) and (39) leads to

$$ \begin{array}{@{}rcl@{}} &&\frac{1}{2}\|x^{k+1}-x^{*}\|_{M_{1}^{k+1}}^{2}+\frac{1}{2}\|z^{k+1}-Ax^{*}\|_{M_{2}^{k+1}+c\text{Id}}^{2}\\ &&+\frac{1}{2c}\|y^{k+1}-y^{*}\|^{2} + \frac{1}{2}\|z^{k+1}-z^{k}\|_{3{M_{2}^{k}}-M_{2}^{k-1}}^{2} \\ & \leq&\frac{1}{2}\|x^{k}-x^{*}\|_{{M_{1}^{k}}}^{2}+\frac{1}{2}\|z^{k}-Ax^{*}\|_{{M_{2}^{k}}+c\text{Id}}^{2}+\frac{1}{2c}\|y^{k}-y^{*}\|^{2}+\frac{1}{2}\|z^{k}-z^{k-1}\|_{M_{2}^{k-1}}^{2}\\ &&-\frac{1}{2}\|x^{k+1}-x^{k}\|_{{M_{1}^{k}}-\frac{L}{2}\text{Id}}^{2} -\frac{c}{2}\|z^{k+1}-z^{k}\|^{2} - \frac{1}{2c}\|y^{k+1}-y^{k}\|^{2}\\ &&-L\left\|L^{-1}\left( \nabla h (x^{*})-\nabla h (x^{k})\right)+\frac{1}{2}\left( x^{k}-x^{k+1}\right)\right\|^{2}. \end{array} $$

Taking into account that, according to Assumption (III), $3{M_{2}^{k}}-M_{2}^{k-1}\succcurlyeq {M_{2}^{k}}$, we can conclude that for all *k* ≥ 1 it holds

56 $$ \begin{array}{@{}rcl@{}} &&\frac{1}{2}\|x^{k+1}-x^{*}\|_{M_{1}^{k+1}}^{2}+\frac{1}{2}\|z^{k+1}-Ax^{*}\|_{M_{2}^{k+1}+c\text{Id}}^{2}\\ &&+\frac{1}{2c}\|y^{k+1}-y^{*}\|^{2} + \frac{1}{2}\|z^{k+1}-z^{k}\|_{{M_{2}^{k}}}^{2}\\& \leq& \frac{1}{2}\|x^{k}-x^{*}\|_{{M_{1}^{k}}}^{2}+\frac{1}{2}\|z^{k}-Ax^{*}\|_{{M_{2}^{k}}+c\text{Id}}^{2}+\frac{1}{2c}\|y^{k}-y^{*}\|^{2}+\frac{1}{2}\|z^{k}-z^{k-1}\|_{M_{2}^{k-1}}^{2}\\ &&-\frac{1}{2}\|x^{k+1}-x^{k}\|_{{M_{1}^{k}}-\frac{L}{2}\text{Id}}^{2}-\frac{c}{2}\|z^{k+1}-z^{k}\|^{2} - \frac{1}{2c}\|y^{k+1}-y^{k}\|^{2}. \end{array} $$

Similarly, in case *L* = 0, we obtain

57 $$ \begin{array}{@{}rcl@{}} &&\frac{1}{2}\|x^{k+1}-x^{*}\|_{M_{1}^{k+1}}^{2}+\frac{1}{2}\|z^{k+1}-Ax^{*}\|_{M_{2}^{k+1}+c\text{Id}}^{2}\\ &&+\frac{1}{2c}\|y^{k+1}-y^{*}\|^{2} + \frac{1}{2}\|z^{k+1}-z^{k}\|_{{M_{2}^{k}}}^{2} \\ & \leq& \frac{1}{2}\|x^{k}-x^{*}\|_{{M_{1}^{k}}}^{2}+\frac{1}{2}\|z^{k}-Ax^{*}\|_{{M_{2}^{k}}+c\text{Id}}^{2}+\frac{1}{2c}\|y^{k}-y^{*}\|^{2}+\frac{1}{2}\|z^{k}-z^{k-1}\|_{M_{2}^{k-1}}^{2}\\ &&-\frac{1}{2}\|x^{k+1}-x^{k}\|_{{M_{1}^{k}}}^{2}-\frac{c}{2}\|z^{k+1}-z^{k}\|^{2} - \frac{1}{2c}\|y^{k+1}-y^{k}\|^{2}. \end{array} $$

Using telescoping sum arguments, we obtain that $\|x^{k+1}-x^{k}\|_{{M_{1}^{k}}-\frac {L}{2}\text {Id}} \rightarrow 0$, $y^{k} - y^{k+1} \rightarrow 0$ and $z^{k} - z^{k+1} \rightarrow 0$ as $k \rightarrow +\infty $. Using (25), it follows that $A(x^{k}-x^{k+1}) \rightarrow 0$ as $k \rightarrow +\infty $, which, combined with the fact that ${M_{1}^{k}}-\frac {L}{2}\text {Id} + A^{*}A \in \mathcal { P}_{\alpha }(\mathcal { {H}})$, for all *k* ≥ 0, yields $x^{k}-x^{k+1} \rightarrow 0$ as $k \rightarrow +\infty $. Consequently, $z^{k} - Ax^{k+1} \rightarrow 0$ as $k \rightarrow +\infty $. Hence, the relations (42)–(45) are fulfilled. On the other hand, from both (56) and (57), we derive

$$ \begin{array}{@{}rcl@{}} &&\exists\lim_{k\rightarrow+\infty}\left( \frac{1}{2}\|x^{k}-x^{*}\|_{{M_{1}^{k}}}^{2}+\frac{1}{2}\|z^{k}-Ax^{*}\|_{{M_{2}^{k}}+c\text{Id}}^{2}\right.\\ &&\qquad\qquad\left.+\frac{1}{2c}\|y^{k}-y^{*}\|^{2}+\frac{1}{2}\|z^{k}-z^{k-1}\|_{M_{2}^{k-1}}^{2}\right). \end{array} $$

By using that

$$ \|z^k-z^{k-1}\|_{M_2^{k-1}}^2 \leq \|z^k-z^{k-1}\|_{M_2^{0}}^2 \leq \|M_2^0\| \|z^k-z^{k-1}\|^2 \ \forall k \geq 1, $$

it follows that $\lim _{k\rightarrow +\infty } \|z^{k}-z^{k-1}\|_{M_{2}^{k-1}}^{2} = 0$, which further implies that (52) holds. From here, the conclusion follows by arguing as in the proof provided in the setting of Assumption (II). □

#### *Remark 13*

Choosing as in Remark 6, ${M_{1}^{k}}= \frac {1}{\tau _{k}}\text {Id} - cA^{*}A$, with *τ*_*k*_ > 0 and such that $\tau :=\sup _{k \geq 0} \tau _{k} \in \mathbb {R}$, and ${M_{2}^{k}} = 0$ for all *k* ≥ 0, we have

$$ \begin{array}{@{}rcl@{}} \left\langle x,\left( M_1^k-\frac{L}{2}\text{Id}\right)x\right\rangle &\geq& \left( \frac{1}{\tau_k}-c\|A\|^2-\frac{L}{2}\right)\|x\|^2\\ &\geq& \left( \frac{1}{\tau}-c\|A\|^2-\frac{L}{2}\right)\|x\|^2 \ \forall x \in \mathcal{ H}, \end{array} $$

which means that under the assumption $\frac {1}{\tau }-c\|A\|^2>\frac {L}{2}$ (which recovers the one in Algorithm 3.2 and Theorem 3.1 in [16]), the operators $M_1^k-\frac {L}{2}\text {Id}$ belong for all *k* ≥ 0 to the class $\mathcal { P}_{\alpha _1}({\mathscr{H}})$, with $\alpha _1:= \frac {1}{\tau }-c\|A\|^2-\frac {L}{2}>0$.

#### *Remark 14*

By taking *h* = 0 and *L* = 0, and in each iteration constant operators $M_1^k = M_1 \succcurlyeq 0$ and $M_2^k = M_2 \succcurlyeq 0$ for all *k* ≥ 0, Theorem 12 in the context of Assumption (I) covers the first situation investigated in [28, Theorem 5.6], where in finite-dimensional spaces the matrix *M*_1_ was assumed to be positive definite and the matrix *M*_2_ to be positive semidefinite.

The arguments used in [28, Theorem 5.6] for proving convergence in the case when *M*_1_ = 0 and *A* has full column rank contain flaws and rely on incorrect statements. Theorem 12 provides in the context of Assumption (III) (for *h* = 0, *L* = 0, $M_1^k=0$ and $M_2^k = M_2 \succcurlyeq 0$ for all *k* ≥ 0) the correct proof of this result.

Finally, we notice that the convergence theorem for the iterates of the classical ADMM algorithm (which corresponds to the situation when *h* = 0, *L* = 0, *M*_1_ = *M*_2_ = 0 and *A* has full column rank, see for example [19]) is covered by Theorem 12 in the context of Assumption (III).
